# HEMA Effects on Autophagy Mechanism in Human Dental Pulp Stem Cells

**DOI:** 10.3390/ma12142285

**Published:** 2019-07-16

**Authors:** Francesca Diomede, Domenico Tripodi, Oriana Trubiani, Jacopo Pizzicannella

**Affiliations:** 1 Department of Medical, Oral and Biotechnological Sciences, University “G. d’Annunzio” Chieti-Pescara, 66100 Chieti, Italy; 2 ASL 02 Lanciano, Vasto, Chieti, “Ss. Annunziata” Hospital, 66100 Chieti, Italy

**Keywords:** autophagy, HEMA, human dental pulp stem cells, p62, Beclin1 ERK, pERK, LC3-I/II

## Abstract

Autophagy is a complex mechanism that permits the degradation of cellular components in order to enhance cell homeostasis, recycling the damaged, dysfunctional, or unnecessary components. In restorative dentistry practice, free resin monomers of 2-hydroxyethyl methacrylate (HEMA) can be released. The aim of this study was to investigate the effect of HEMA on proliferation and autophagy in human dental pulp stem cells (hDPSCs). Human DPSCs were treated with different concentrations of HEMA (3 and 5 mmol L^−1^). To evaluate the proliferation rate, MTT and trypan blue assays were used. Autophagic markers such as microtubule-associated protein 1 light chain 3 (LC3-I/II) and ubiquitin-binding protein (p62) were analyzed through immunofluorescence observations. Beclin1, LC3-I/II, and p62 were evaluated by means of Western blotting detection. Considering that activity of extracellular signal–regulated kinase (ERK) and its phosphorylated form (pERK) mediates several cellular processes, such as apoptosis, autophagy, and senescence, the involvement of ERK/pERK signaling was also evaluated. Obtained results showed a decreased cell proliferation associated with morphological changes in HEMA-treated cells. The Western blot results showed that the expression levels of Beclin1, LC3-I/II, and ERK were significantly elevated in HEMA-treated cells and in cells co-treated with rapamycin, an autophagic promoter. The expression levels of p62 were significantly reduced compared to the untreated samples. Protein levels to the autophagic process, observed at confocal microscopy confirmed the data obtained from the Western blot. The up-regulation of ERK and pERK levels, associated with nuclear translocation, revealed that ERK pathway signaling could act as a promoter of autophagy in dental pulp stem cells treated with HEMA.

## 1. Introduction

In restorative dentistry, resin-based dental materials are widely used to restore tooth damage. New generations of this material contain a large quantity of methacrylate monomers, such as 2,2-bis [4-(2-hydroxy-3-methacrylyloxy-propoxy)phenyl] propane (bis-GMA), urethane dimethacrylate (UDMA), hydrophilic monomers such as 2-hydroxyethyl methacrylate (HEMA), and triethylene glycol dimethacrylate (TEGDMA) [1]. Methacrylate agents can penetrate through dentinal tubules and affect the dental pulp tissue, which has led to major clinical disadvantages [2]. Monomers derived from resin-based materials could exert cytotoxic effects on odontoblasts, due to an incomplete polymerization during clinical practice [3,4,5]. This phenomenon had a negative impact on biocompatibility, also inducing the release of pro-inflammatory cytokines. It has been demonstrated that HEMA 3 and 5 mmol L^−1^ can induce an up-regulation of interleukin (IL) 6, IL8, monocyte chemoattractant protein-1 (MCP1), and interferon gamma (IFNɣ) inflammatory molecule secretion [6].

It has been demonstrated that the use of HEMA and TEGDMA delays the odontogenic differentiation process of stem cells derived from dental pulp tissue. Moreover, these monomers negatively affect the mineralization of apical papilla stem cells and induce the secretion of inflammatory cytokines [7,8]. Odontoblasts are cells that form and maintain dentin tissue homeostasis. They have a neural crest origin, and are localized in the periphery of the pulp tissue; during tooth formation, they secrete the organic dentin matrix, while during tooth whole life, they are responsible for the production of secondary and tertiary dentin. A complex autophagic–lysosomal system regulates their lifespan in order to guarantee organelle and protein renewal [9]. 

Autophagy is an intracellular process that degrades organelles or cellular components in order to ensure the maintenance of cell homeostasis. It can be considered a genetically programmed, adaptive response to stress [10,11]. 

Previously, it has been described that HEMA treatment clearly induced autophagy in human fibroblasts [12]. The recent discovery of stem cells in dental pulp has offered the opportunity to study a relevant cellular model from a cell population homologous to the primary tissue, and representing an in vitro model to understand the processes occurring during in vivo HEMA treatment. 

Autophagy is a complex process that can affect cellular properties using different pathways and involving different molecules, such as microtubule-associated protein 1 light chain 3 (LC3-I/II), (ubiquitin-binding protein) p62, and Beclin1. 

LC3 is a soluble protein involved in the autophagic mechanism stimulating the formation of the autophagosome. Two forms of LC3 exist: LC3-I, localized at the cytoplasmic level; and LC3-II, which is a converted form of LC3-I conjugated with phosphatidylethanolamine, localized in the internal and external compartments of the autophagosome. During the autophagic process, LC3-II is degraded in the autolysosomal lumen. In this way, lysosomal turnover of the autophagosomal marker LC3-II reflects HEMA-induced autophagic activity; the assessment of LC3 by immunoblotting or immunofluorescence can therefore be a suitable approach in evaluation of autophagy processes [13].

P62/SQSTM1, known as sequestosome-1, is involved in several cellular actions, in particular, nuclear localization signals other than an LC3-interacting region (LIR) [14]. Measuring the P62 level is considered a common method for monitoring autophagic flux. In fact, p62 represents an autophagy substrate, and it plays a key role in regulation of the degradation of damaged cell components [15]. Its activity is involved in different diseases other than the adipogenesis process, interacting with several signaling pathways, including Kelch-like ECH-associated protein 1–nuclear factor-like 2 (Keap1-Nrf2) [16], mammalian target of rapamycin (m-TOR) [17,18], and nuclear factor kappa-light-chain-enhancer of activated B cells (NF-κB) [19]. 

Autophagy is also regulated by ERK and pERK signaling, proteins that are related to the family of mitogen-activated protein kinases (MAPKs) [20,21]. 

**Figure 1:**
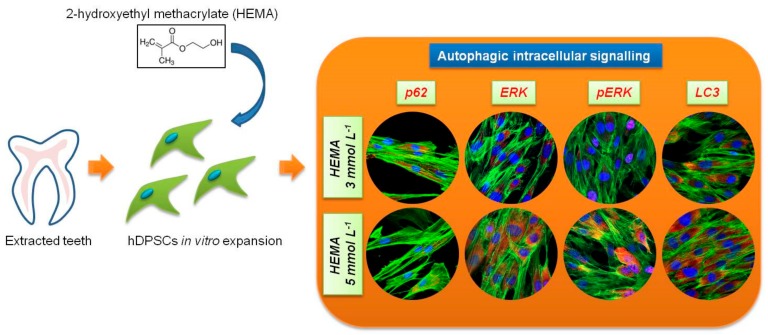
Schematic view of the experimental design. hDPSCs, human Dental Pulp Stem Cells.

These types of transduction intracellular signal cascade are activated through phosphorylation, which induces a nuclear translocation [20]. The role of MEK/ERK in autophagy activation has been reported in the literature. MEK/ERK signaling mediates autophagy, involving several mechanisms, such as amino acid deprivation [22], aurintricarboxylic acid [23], B-group soyasaponins [24], and curcumin [25,26,27]. Beclin-1 is regulated by MEK/ERK signaling to stimulate the autophagy process. The aim of this study was to analyze the expression of LC3-I/II and p62 specific autophagic markers and correlate the regulatory mechanisms of autophagy to p38MAPK/ERK and pERK pathway signal in human dental pulp stem cells after 24 h of HEMA treatment at various concentration (Figure 1).

## 2. Materials and Methods

### 2.1. Cell Culture

The Ethics Committee of the University of Chieti approved the research study (n°266/University of Chieti). To obtain hDPSCs, dental pulp was collected by a procedure previously reported [28]. Dental pulp was collected from human teeth scheduled to be removed for orthodontic therapy. Explants were cultured in petri dishes with mesenchymal stem cells growth medium-chemically defined (MSCGM-CD) (Lonza, Basel, Switzerland) [29]. Three times a week, the medium was replaced with fresh medium. After two weeks in culture, cells spontaneously migrated from explants. All experiments were performed with cells at second passage.

### 2.2. Cell Characterization

To characterize mesenchymal stem cells, cytofluorimetric analysis has been performed as previously described [30]. Cells were assessed for the positivity or negativity of Sox-2, Oct3/4, CD13, C14, CD29, CD34, CD45, CD73, CD90, and CD105. The following antibodies were used for cell characterization: fluorescein isothiocyanate-conjugated anti-CD13 (CD13 FITC), phycoerythrin-conjugated anti-CD29 (CD29 PE), FITC-conjugated anti-CD45 (CD45 FITC), and anti-CD105 (CD105 FITC), were obtained from Ancell; FITC-conjugated anti-CD14 (CD14 FITC) was purchased from Milteny Biotec; PE-conjugated anti-CD73 (CD73 PE), FITC-conjugated anti-CD90 (CD90 FITC), Alexa488-conjugated anti-Sox2 (Sox2 Alexa488), FITC-conjugated anti-SSEA-4 (SSEA-4 FITC), and PE-conjugated anti-OCT3/4 (OCT3/4 PE) were obtained from Becton Dickinson; PE-conjugated anti-CD34 (CD34-PE) was purchased from Beckman Coulter; and an appropriate secondary FITC-conjugated antibody was obtained from Jackson Immunoresearch Laboratories. Washing buffer (phosphate-buffered saline, PBS, 0.1% sodium azide, and 0.5% bovine serum albumin, BSA) was used for all washing steps (3 mL of washing buffer and centrifugation, 400× *g* for 8 min at 4 °C). Briefly, 5 × 10^5^ cells/sample were incubated with 100 mL of 20 mM ethylenediaminetetraacetic acid (EDTA) at 37 °C for 10 min and washed. Staining of surface antigens and intracellular antigens was carried out according to Trubiani et al. [30]. Quality control included a regular check-up with Rainbow Calibration Particles (BD Biosciences). Debris was excluded from the analysis by gating on morphological parameters; 20,000 non-debris events in the morphological gate were recorded for each sample. To assess nonspecific fluorescence, we used specific irrelevant controls. All antibodies were titrated under assay conditions, and optimal photomultiplier (PMT) gains were established for each channel. Data were analyzed using FlowJosoftware (TreeStar). Mean fluorescence intensity ratio (MFI Ratio) was calculated by dividing the MFI of positive events by the MFI of negative events.

To evaluate their capacity to adhere to the plastic substrate, cells were fixed and the toluidine blue solution was then used to stain hDPSCs in order to evaluate cell morphology using inverted light microscopy [31].

For osteogenic differentiation, the cells were cultured in osteogenic differentiation medium kit (Lonza). The cells were stained with Alizarin red S solution after 21 days of induction [32]. Adipogenic differentiation was induced by culturing the hDPSCs in adipogenic medium kit (Lonza) for 28 days, and the cells were assessed using Adipo Oil red O staining solution [33].

Osteogenesis and adipogenesis commitment were confirmed by real-time PCR to evaluate the expression of Runt-related transcription factor 2 (RUNX2), alkaline phosphatase (ALP), fatty acid binding protein-4 (FABP4), and peroxisome proliferator-activated receptor-ɣ (PPARɣ) [34]. 

Total RNA was isolated from differentiated and undifferentiated hDPSCs using the RNeasy Plus Universal Mini Kit (Qiagen, Valencia, CA, USA). The ABI PRISM 7900 HT Sequence Detection System (Applied Biosystems, Foster City, CA, USA) was used for qPCR of studied markers (ALP Hs01029144_m1; RUNX2 Hs00231692_m1; FABP4 Hs01086177_m1; PPARɣ Hs01115513_m; Applied Biosystems). Beta-2 microglobulin (B2M, Hs99999907_m1; Applied Biosystems) was used for template normalization [35]. Comparative 2^−ΔΔCt^ relative quantification method was used to analyze the mRNA expression. 

### 2.3. Cell Proliferation and Viability Assay

Cell proliferation was evaluated through MTT assay, as previously reported [36]. A total 2 × 10^3^ cells per well were seeded into 96 well plates in 200 μL of medium to test all considered conditions: hDPSCs, hDPSCs treated with HEMA 3 mmol L^−1^, and hDPSCs treated with HEMA 5 mmol L^−1^ for 24 h. At different endpoints—24, 48, and 72 h—20 μL of MTT (Promega, Milan, Italy) solution was added to each well. Absorbance at 490 nm was measured with a reference wavelength of 630 nm [37].

Cell viability was assessed by trypan blue exclusion test. For this purpose, all samples were incubated with trypan blue solution at the same endpoints used for the MTT test (24, 48, and 72 h) and subsequently analyzed by light microscopy, using a Burker’s chamber as previously described [38]. 

### 2.4. Immunohistochemistry and Confocal Laser Scanning Microscope (CLSM) Analysis

For immunofluorescence detection, cells grown on eight well chamber slides were fixed using 4% paraformaldehyde, diluted in 0.1M sodium phosphate buffer (PBS, Lonza) [39]. After the fixation step, cells were permeabilized with 0.5% Triton X-100 in PBS for 10 min, followed by blocking with 5% skimmed milk in PBS for 30 min [40]. Primary antibodies used for immunofluorescence were purchased from Santa Cruz Biotechnology (Santa Cruz Biotechnology, Santa Cruz, CA, USA). P62 (1:200, Santa Cruz Biotechnology, Santa Cruz, CA, USA), ERK (1:100, Santa Cruz Biotechnology), pERK (1:100, Santa Cruz Biotechnology), and LC3 (1:250, Santa Cruz Biotechnology) were used as primary antibodies. Cells were then incubated with Alexa Fluor 568 red fluorescence conjugated goat anti-rabbit as secondary antibodies (1:200, Molecular Probes, Invitrogen, Eugene, OR, USA). Alexa Fluor 488 phalloidin green fluorescence conjugate (1:400, Molecular Probes) was used to mark the actin cytoskeleton. After immunofluorescence labeling, cells were washed and incubated with TOPRO (1:200, Molecular Probes) for 1 h at 37 °C [41]. Samples were observed using a Zeiss LSM800 confocal system (Zeiss, Jena, Germany). The relative fluorescence intensities of p62, ERK, pERK, and LC3 were quantified using NIS-Elements AR imaging software (Nikon). For the counting statistics of immunofluorescence-positive nuclei for ERK and pERK, ten views (100×) were randomly chosen in each experimental group and analyzed using NIS-Elements AR imaging software (Nikon). All the experiments were repeated at least three times. Data are presented as the mean and standard error of the mean (mean ± SEM). The comparison analysis of different groups was done using a one-way analysis of variance followed by a post hoc Bonferroni evaluation using GraphPad Prism5. Differences were termed statistically significant at *p* < 0.05.

### 2.5. Western Blot Analysis

Proteins (30 μg) derived from treated and untreated hDPSCs with and without 0.1 µL of rapamycin (Sigma Aldrih, Milan, Italy) were processed as previously described [42]. All antibodies used for the Western blot procedure were purchased from Santa Cruz Biotechnology. After protein separation, saturated sheets were incubated overnight at 4 °C with P62 (1:200, Santa Cruz Biotechnology), LC3-I/II (1:1000, Santa Cruz Biotechnology), ERK (1:750, Santa Cruz Biotechnology), pERK (1:750, Santa Cruz Biotechnology), Beclin1 (1:1000, Santa Cruz Biotechnology), and β-actin (1:1000, Santa Cruz Biotechnology).

Samples were then washed and incubated in secondary antibody diluted 1:1000 in 1× TBS, 5% milk, 0.05% Tween-20. Protein-specific bands were visualized via the electrochemiluminescence (ECL) method [43]. 

### 2.6. Statistical Analysis

Graph Pad Prism 6.0 (GraphPad Software, La Jolla, CA, USA) was used to perform the statistical evaluation. Student’s *t*-test was used to assess the differences between the groups. Obtained results are reported as mean ± SEM. A *p*-value < 0.05 was considered statistically significant.

## 3. Results

### 3.1. Characterization of hDPSCs

**Figure 2:**
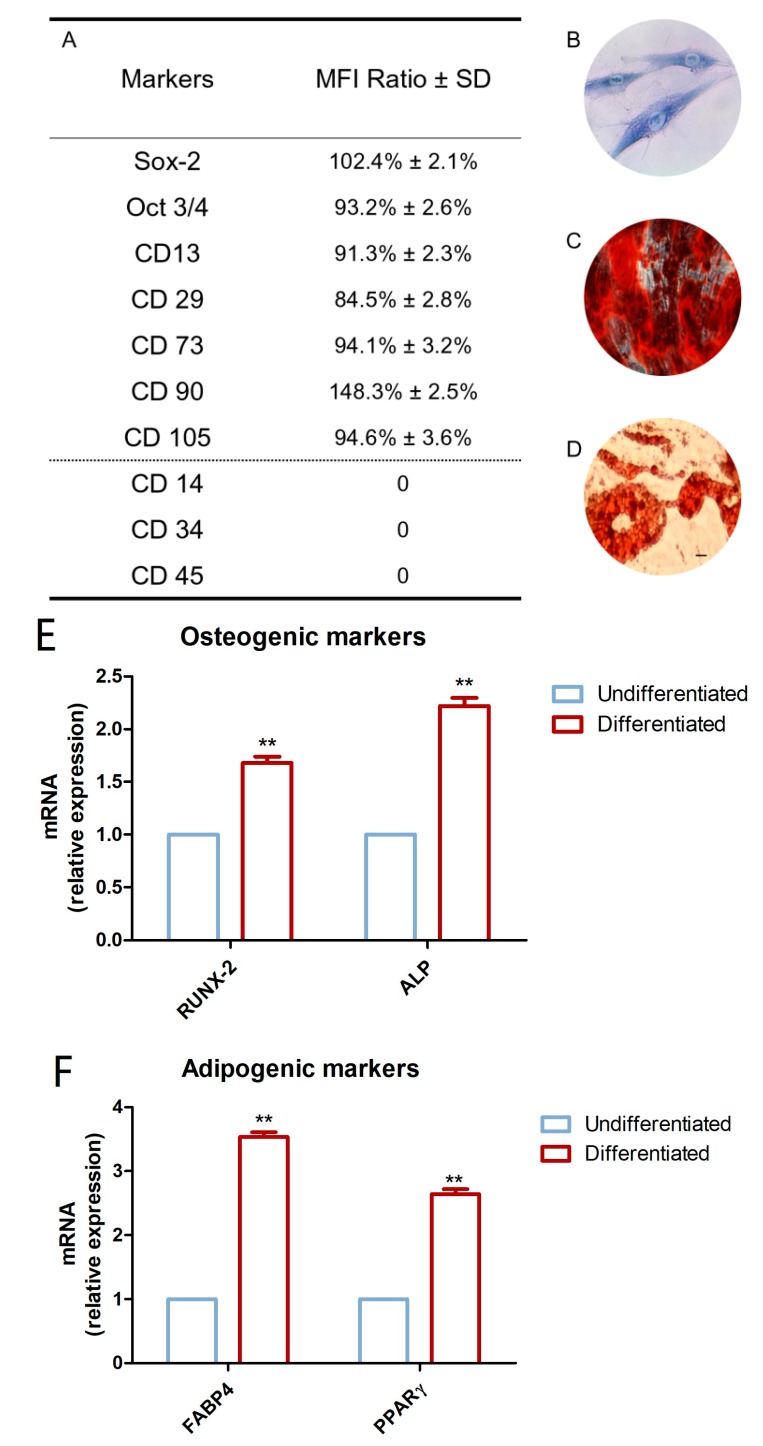
Huma Dental Pulp Stem Cells (hDPSC) characterization. (**A**) Cytofluorimetric detection of hDPSCs at second passage. Median Fluorescence Intensity (MFI) ratio is the average of five different biological samples ± standard deviation (SD); cutoff ratio positivity > 2.0. (**B**) Plastic-adherent hDPSCs observed by inverted light microscopy, stained with toluidine blue solution. (**C**) Osteogenic differentiated hDPSCs stained with Alizarin Red S solution. (**D**) Adipogenic differentiated hDPSCs stained with Adipo Oil red O solution. (**E**) Real Time-Polimerase Chain Reaction (RT-PCR) of Runt-related transcription factor 2 (RUNX2) and Alkaline Phosphatase (ALP) performed in undifferentiated and differentiated hDPSCs. (**F**) RT-PCR of Fatty Acid-Binding Protein 4 (FABP4) and Peroxisome Proliferator-Activated Receptor Gamma (PPARɣ) performed in undifferentiated and differentiated hDPSCs. Scale bar: 10 µm. ** *p* < 0.01.

To characterize the hDPSC profiles, cytofluorimetric analysis and mesengenic differentiation ability were evaluated after cell isolation. The phenotype profile of the hDPSCs was determined by cytofluorimetric analysis. Human DPSCs showed a positivity for the following markers: Sox-2, Oct3/4, CD13, CD29, CD73, CD90, and CD105, while they were negative for C14, CD34, and CD45 (Figure 2A). 

Plastic-adherent cells showed a fibroblastic morphology with long cytoplasmic processes and an evident nucleus (Figure 2B). 

The capacity of hDPSCs to differentiate into osteoblasts and adipoblasts has been demonstrated through in vitro experiments. Alizarin Red S solution staining has been used to assess osteogenic ability; for this purpose, cells were stained within 21 days of the induction period. Calcium deposits were detected in red by inverted light microscopy (Figure 2C). Adipo Oil red O staining was used to evaluate the hDPSCs’ capacity to differentiate towards an adipogenic phenotype. Cells were maintained under adipogenic conditions, and after 28 days of culture, lipid vacuoles at the cytoplasmic level were evident by inverted light microscopy observation (Figure 2D). Gene expression was assessed to confirm the qualitative data obtained at light microscopy. RT-PCR was carried out on undifferentiated and differentiated samples. Differentiated hDPSCs showed an up-regulation in the mRNA expression of RUNX2 and ALP in cells maintained under osteogenic conditions when compared to the undifferentiated cells (Figure 2E). Human DPSCs cultured under adipogenic conditions showed an up-regulation in the expression of FABP4 and PPARɣ (Figure 2F).

### 3.2. HEMA Effects on Cell Proliferation Rate

**Figure 3:**
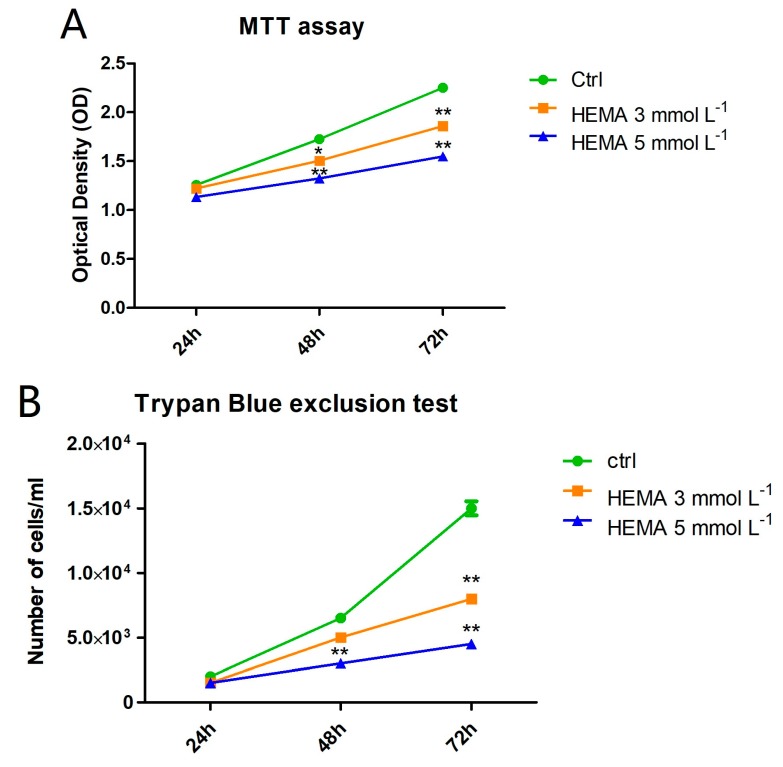
Cell viability and proliferation. (**A**) The results of MTT assay in untreated hDPSCs and in hDPSCs treated with HEMA 3 mmol L^−1^ and HEMA 5 mmol L^−1^. (**B**) The results of Trypan Blue test in untreated hDPSCs and in hDPSCs treated with HEMA 3 mmol L^−1^ and HEMA 5 mmol L^−1^. * *p* < 0.05, ** *p* < 0.01.

To evaluate cell proliferation and viability, MTT and Trypan blue assays were performed on all considered samples. Cells treated with HEMA 3 mmol L^−1^ and HEMA 5 mmol L^−1^ showed a lower proliferation rate from 24 to 72 h of culture when compared to the untreated cells (Figure 3A). The same trend has been demonstrated in the Trypan blue line graph (Figure 3B).

### 3.3. Autophagic Marker Expression

**Figure 4:**
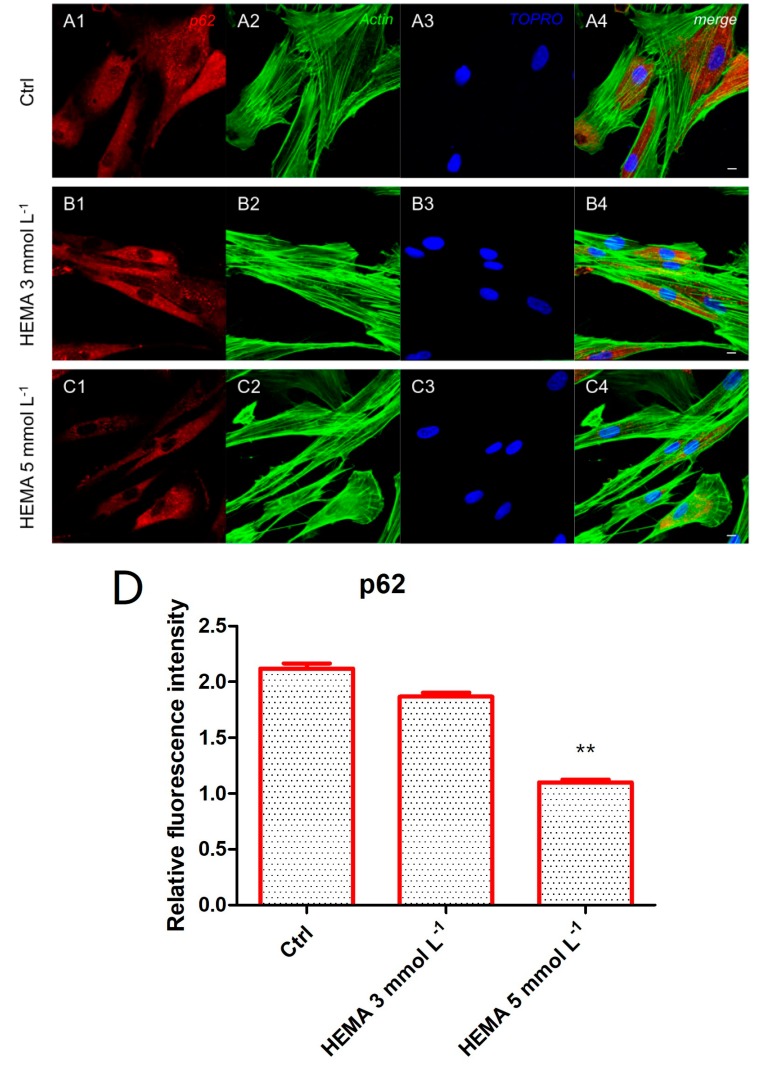
p62 expression. (**A1**) Untreated cells showed a strong positive expression for p62 marker. (**A2**) Actin cytoskeleton in untreated hDPSCs. (**A3**) Nuclei in untreated hDPSCs. (**A4**) Merged picture of abovementioned channels in untreated hDPSCs. (**B1**) Cells treated with HEMA 3 mmol L^−1^ showed a positive expression for p62 marker. (**B2**) Cytoskeleton actin in HEMA 3 mmol L^−1^ p62 treated hDPSCs. (**B3**) Nuclei in HEMA 3 mmol L^−1^ treated hDPSCs. (**B4**) Merged picture of abovementioned channels in HEMA 3 mmol L^−1^ treated hDPSCs. (**C1**) Cells treated with HEMA 5 mmol L^−1^ showed a low positive expression for p62 marker. (**C2**) Actin cytoskeleton in HEMA 5 mmol L^−1^ treated hDPSCs. (**C3**) Nuclei in HEMA 5 mmol L^−1^ treated hDPSCs. (**C4**) Merged picture of abovementioned channels in HEMA 5 mmol L^−1^ treated hDPSCs. (**D**) The relative fluorescent intensity of p62 was analyzed using NIS-Elements AR imaging software. Data are presented as the mean of thirty measurements ± standard deviation. ** *p* < 0.01. Green fluorescence: actin cytoskeleton, red fluorescence: p62, blue fluorescence: nuclei. Magnification: 63×. Scale bar: 10 µm.

**Figure 5:**
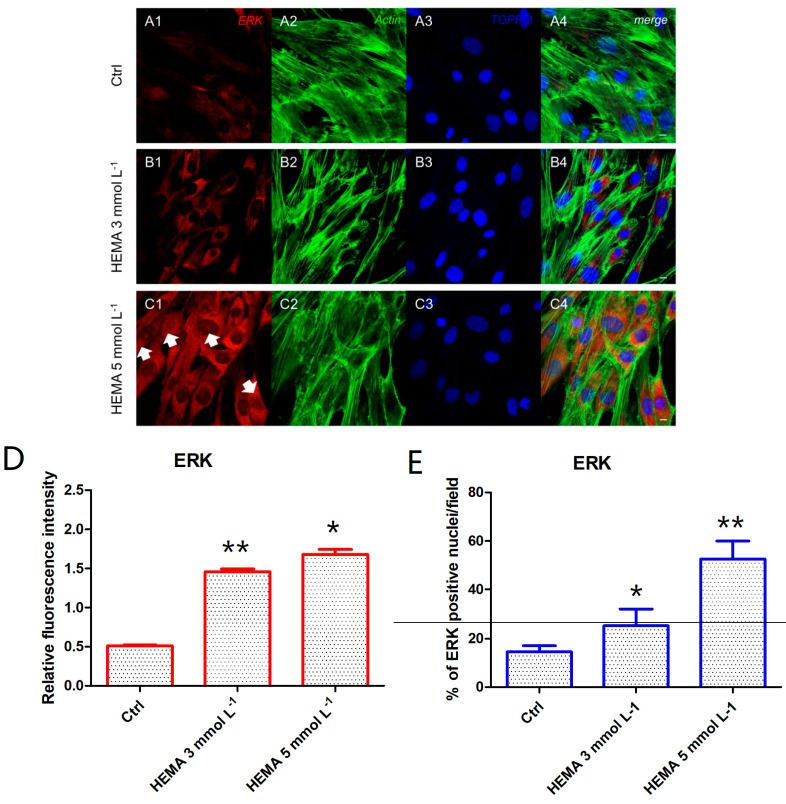
ERK expression. (**A1**) Untreated cells showed a low positive expression for ERK. (**A2**) Actin cytoskeleton in untreated hDPSCs. (**A3**) Nuclei in untreated hDPSCs. (**A4**) Merged picture of abovementioned channels in untreated hDPSCs. (**B1**) Cells treated with HEMA 3 mmol L^−1^ showed a positive expression for ERK. (**B2**) Actin cytoskeleton in HEMA 3 mmol L^−1^ treated hDPSCs. (**B3**) Nuclei in HEMA 3 mmol L^−1^ treated hDPSCs. (**B4**) Merged picture of abovementioned channels in HEMA 3 mmol L^−1^ treated hDPSCs. (**C1**) Cells treated with HEMA 5 mmol L^−1^ showed a strong positive expression for ERK, with a nuclear localization (arrows). (**C2**) Actin cytoskeleton in HEMA 5 mmol L^−1^ treated hDPSCs. (**C3**) Nuclei in HEMA 5 mmol L^−1^ treated hDPSCs. (**C4**) Merges picture of abovementioned channels in HEMA 5 mmol L^−1^ treated hDPSCs. (**D**) The relative fluorescent intensity of ERK was analyzed using NIS-Elements AR imaging software. Data are presented as the mean of 30 measurements ± standard deviation. (**E**) Analysis of the percentage of ERK-positive nuclei/field. * *p* < 0.05; ** *p* < 0.01. Green fluorescence: actin cytoskeleton, red fluorescence: ERK, blue fluorescence: nuclei. Magnification: 63×. Scale bar: 10 µm.

**Figure 6:**
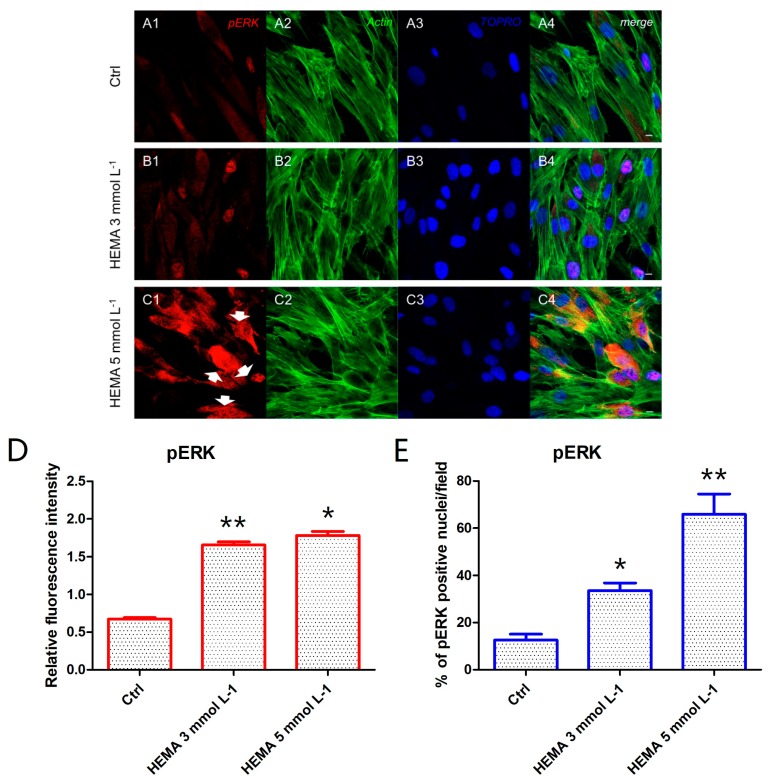
pERK expression. (**A1**) Untreated cells showed a low positive expression for pERK. (**A2**) Actin cytoskeleton in untreated hDPSCs. (**A3**) Nuclei in untreated hDPSCs. (**A4**) Merged picture of abovementioned channels in untreated hDPSCs. (**B1**) Cells treated with HEMA 3 mmol L^−1^ showed a positive expression for pERK. (**B2**) Actin cytoskeleton in HEMA 3 mmol L^−1^ treated hDPSCs. (**B3**) Nuclei in HEMA 3 mmol L^−1^ treated hDPSCs. (**B4**) Merged picture of abovementioned channels in HEMA 3 mmol L^−1^ treated hDPSCs. (**C1**) Cells treated with HEMA 5 mmol L^−1^ showed a strong positive expression for pERK, with a nuclear localization (arrows). (**C2**) Actin cytoskeleton in HEMA 5 mmol L^−1^ treated hDPSCs. (**C3**) Nuclei in HEMA 5 mmol L^−1^ treated hDPSCs. (**C4**) Merged picture of abovementioned channels in HEMA 5 mmol L^−1^ treated hDPSCs. (**D**) The relative fluorescent intensity of pERK was analyzed using NIS-Elements AR imaging software. Data are presented as the mean of 30 measurements ± standard deviation. (**E**) Analysis of the percentage of pERK-positive nuclei/field. * *p* < 0.05; ** *p* < 0.01. Green fluorescence: actin cytoskeleton, red fluorescence: pERK, blue fluorescence: nuclei. Magnification: 63×. Scale bar: 10 µm.

**Figure 7:**
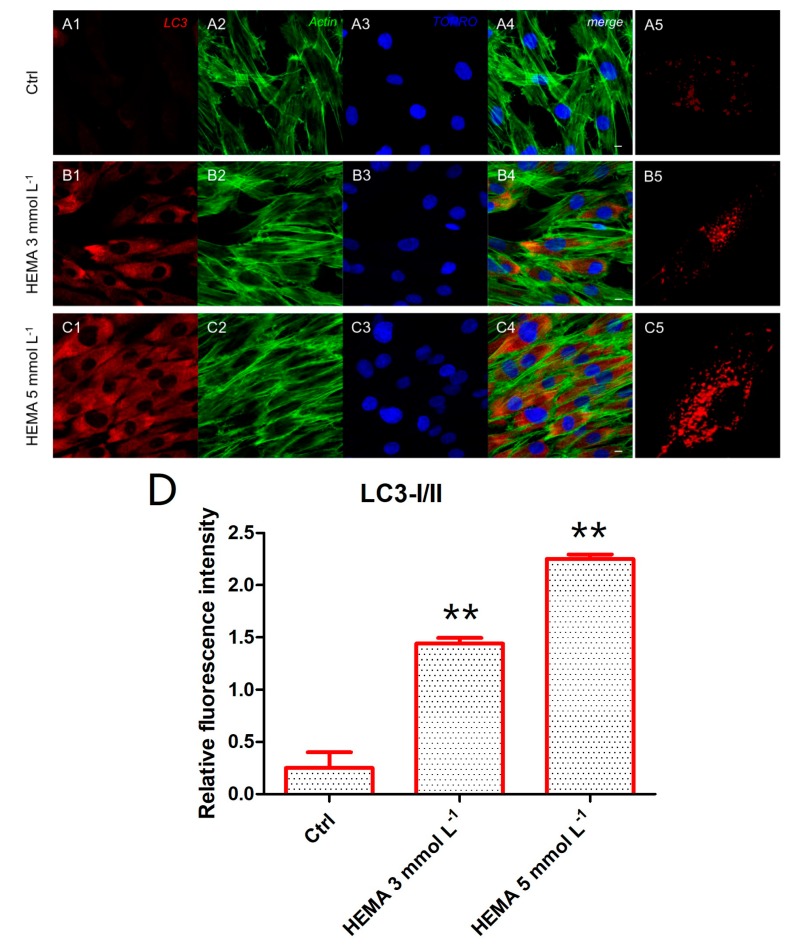
LC3 expression. (**A1**) Untreated cells showed a negative expression for LC3 marker. (**A2**) Actin cytoskeleton in untreated hDPSCs. (**A3**) Nuclei in untreated hDPSCs. (**A4**) Merged picture of abovementioned channels in untreated hDPSCs. (**A5**) Untreated hDPSCs showed a low positivity for LC3 at high magnification (100×). (**B1**) Cells treated with HEMA 3 mmol L^−1^ showed a positive expression for LC3 marker. (**B2**) Actin cytoskeleton in HEMA 3 mmol L^−1^ treated hDPSCs. (**B3**) Nuclei in HEMA 3 mmol L^−1^ treated hDPSCs. (**B4**) Merged picture of abovementioned channels in HEMA 3 mmol L^−1^ treated hDPSCs. (**B5**) hDPSCs treated with HEMA 3 mmol L^−1^ showed a positivity for LC3 at high magnification (100×). (**C1**) Cells treated with HEMA 5 mmol L^−1^ showed a strong positive expression for LC3 marker. (**C2**) Actin cytoskeleton in HEMA 5 mmol L^−1^ treated hDPSCs. (**C3**) Nuclei in HEMA 5 mmol L^−1^ treated hDPSCs. (**C4**) Merged picture of abovementioned channels in HEMA 5 mmol L^−1^ treated hDPSCs. (**C5**) hDPSCs treated with HEMA 5 mmol L^−1^ showed a positivity for LC3 at high magnification (100×). (**D**) The relative fluorescent intensity of LC3 was analyzed using Nikon Isnstrument Software (NIS)-Elements Advanced Research imaging software. Data are presented as the mean of 30 measurements ± standard deviation. ** *p* < 0.01. Green fluorescence: actin cytoskeleton, red fluorescence: LC3, blue fluorescence: nuclei. Magnification: 63×. Scale bar: 10 µm.

Immunofluorescence staining was performed to evaluate the expression of markers related to the autophagic mechanism. P62 showed a decreased expression in hDPSCs treated with HEMA 3 mmol L^−1^ and HEMA 5 mmol L^−1^ compared to the untreated cells, observed by confocal microscopy (Figure 4A1–C4). LC3 and ERK showed an opposite regulation, untreated cells showed a negative expression of LC3 and a light positivity for ERK. As shown in Figure 5C1,C4, hDPSCs were positive to ERK, demonstrating a nuclear translocation after HEMA 5 mmol L^−1^ treatment. The phosphorylated form of ERK showed the same trend of ERK expression, as demonstrated by the percentage of nuclear localization of pERK in HEMA 5 mmol L^−1^ treated cells when compared to untreated (Figure 6E). In hDPSCs treated with HEMA 3 mmol L^−1^ and HEMA 5 mmol L^−1^, LC3 showed a high expression localized at the cytoplasmic level (Figure 7A1–C4). 

### 3.4. Autophagic Marker Levels

**Figure 8:**
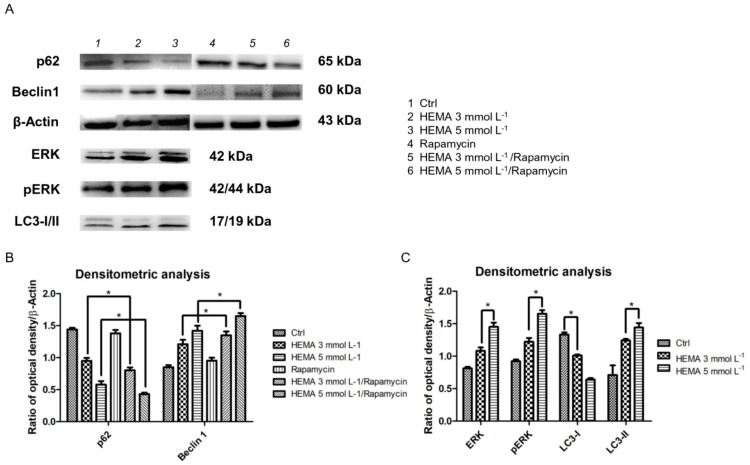
Western blot analyses. (**A**) Specific bands of p62, Beclin1, ERK, pERK, LC3-I/II, and β-Actin, used as housekeeping protein. (**B**) Densitometric analysis histograms of p62 and Beclin1 in all experimental groups untreated and treated with rapamycin. (**C**) Densitometric analysis histograms of ERK, pERK, and LC3-I/II in untreated hDPSCs and treated with HEMA 3 mmol L^−1^ and HEMA 5 mmol L^−1^. * *p* < 0.01.

Beclin-1 and P62 are classical autophagic markers. To evaluate their expression, Western blot analyses were performed. The protein levels of p62 decreased in HEMA 3 and 5 mmol L^−1^ treated samples, while Beclin1-specific protein bands showed an up-regulation in treated cells (Figure 8A). In particular, the results were enhanced in samples treated with rapamycin, an autophagic inductor (Figure 8B).The expression of ERK, pERK, and LC3-I/II was increased in HEMA 3 and 5 mmol L^−1^ treated cells when compared to the control (*p* < 0.05). All experiments were repeated in triplicate. (Figure 8C).

## 4. Discussion

Methacrylate-based restorative materials are common materials used in clinical practice to restore tooth damage, function, and at the same time ensure the desired aesthetic component. These materials are commercialized in a viscous form to be better manipulated during clinical practice, and they are then subjected to a polymerization process to be converted into their solid form [44]. Frequently, an incomplete polymerization can induce the release of monomers into the oral cavity, which move towards dentin micro-channels to enter the vascular system, inducing an inflammatory response, causing an alteration to the odontoblastic function and subsequent pulp tissue damage.

HEMA has been reported to alter the homeostasis of various cell lines in vitro, inducing DNA damage, apoptosis, and necrosis and autophagy [12].

Autophagy is an intracellular catabolic process that preserves cell homeostasis, involving the degradation of damaged organelles and/or toxic macromolecules. Autophagy machinery plays a direct or indirect role in health and disease by way of lysosomal activity. Mitophagy could be considered a selective autophagy mechanism that induces the degradation of mitochondria in response to damage or stress. Damaged mitochondria produce high levels of reactive oxygen species (ROS) that trigger the mitophagy process [26]. Autophagy has a dual role in tumor cells, acting as a tumor suppressor and, at the same time, enhancing tumor cell growth. Autophagy also plays a key role in the suppression or not of inflammatory processes. Meanwhile, inflammatory processes can induce or block the autophagy pathway.

The main goal of this work was to demonstrate the involvement of autophagy in response to low concentrations 3 and 5 mmol L^−1^ of HEMA treatment in hDPSCs as an adaptive machinery to ensure cell homeostasis. For this purpose, the capacity of LC3, p62, and ERK signaling to counteract on the control of autophagy induced by HEMA treatment in stem cells from dental pulp was evaluated. Our findings provided evidence that hDPSCs express stemness markers [30], and are competent to differentiate into osteoblast and adipoblast lineages [27].

Colorimetric detection to assess cell metabolic activity (MTT assay) and the Trypan blue exclusion test to determine the number of viable cells provided evidence that the exposure to 3 and 5 mmol L^−1^ of HEMA induced lower growth relative to untreated cells, from 24 h to 72 h of treatment. The reduction of cell proliferation and the change in classical cell morphology can thus be directly associated with the HEMA treatment [5]. This research paper focused on metabolic changes that occur in the first 24 h of treatment. In particular, the expression at the confocal microscopy level and Western blotting analyses of autophagic markers such as LC3 and p62, in addition to the molecular signaling pathway ERK, were evaluated.

These types of intracellular signal transduction cascades are activated through phosphorylation that induces nuclear translocation [20]. The role of MEK/ERK in autophagy activation has been reported in the literature. MEK/ERK signaling mediates autophagy, involving several mechanisms, including amino acid deprivation [22], aurintricarboxylic acid [23], B-group soyasaponins [24], and curcumin [25]. Beclin-1 has been shown to be regulated by MEK/ERK signaling to stimulate the autophagy process [16]. In this study, we have provided evidence that after 24 h of 3 and 5 mmol L^−1^ HEMA treatment, an up-regulation of LC3 in parallel with a p62 down-regulation was detected. Moreover, a significant increase of ERK protein level was demonstrated, together with its nuclear translocation in treated samples, demonstrating that HEMA treatment induced an autophagic process through the positive modulation of ERK signaling. Beclin-1 is the first mammalian gene found to mediate autophagy, such as regulating the turnover of proteins controlling the growth and proliferation of cells [45]. Our results demonstrated a decreased level of p62 in HEMA treated cells and in HEMA + rapamycin treated samples and at the same time we highlighted a overexpression of Beclin1 in HEMA treated cells, more pronounced in samples treated with HEMA + rapamycin treated hDPSCs showed a decrease of p62 and an overexpression of Beclin1 when compared to untreated hDPSCs. ERK, pERK, and LC3-II showed an upregulation in 3 and 5 mmol L^−1^ HEMA when compared to control cells, while LC3-I showed an opposite regulation.

Rapamycin is a special prophylactic for the mammalian target of rapamycin (mTOR), which binds fk506-binding protein 12 kDa (FKBP12) to form a molecular complex that inhibits mTOR activity [46], and is considered an autophagy promoter. We also evaluated the influence of rapamycin on the expression of classical autophagic markers in treated and untreated hDPSCs. In this study, Western blot analysis indicated that rapamycin increased Beclin1 levels, but decreased p62 levels. In response to HEMA injury, dental pulp stem cells activate autophagy as a pro-survival cytoprotective mechanism. Further studies are necessary to consider the strategic and therapeutic applications of this research in tissue repair and regeneration.

## References

[1] Diomede F., Caputi S., Merciaro I., Frisone S., D’Arcangelo C., Piattelli A., Trubiani O. (2014). Pro-inflammatory cytokine release and cell growth inhibition in primary human oral cells after exposure to endodontic sealer. Int. Endod. J..

[2] Munoz-Bonilla A., Lopez D., Fernandez-Garcia M. (2018). Providing antibacterial activity to poly(2-hydroxy ethyl methacrylate) by copolymerization with a methacrylic thiazolium derivative. Int. J. Mol. Sci..

[3] Bakopoulou A., Papadopoulos T., Garefis P. (2009). Molecular toxicology of substances released from resin-based dental restorative materials. Int. J. Mol. Sci..

[4] Spagnuolo G., D’Anto V., Cosentino C., Schmalz G., Schweikl H., Rengo S. (2006). Effect of n-acetyl-l-cysteine on ros production and cell death caused by hema in human primary gingival fibroblasts. Biomaterials.

[5] Schweikl H., Hartmann A., Hiller K.A., Spagnuolo G., Bolay C., Brockhoff G., Schmalz G. (2007). Inhibition of tegdma and hema-induced genotoxicity and cell cycle arrest by n-acetylcysteine. Dent. Mater. Off. Publ. Acad. Dent. Mater..

[6] Trubiani O., Ballerini P., Murmura G., Pizzicannella J., Giuliani P., Buccella S., Caputi S. (2012). Toll-like receptor 4 expression, interleukin-6,-8 and ccl-20 release, and nf-kb translocation in human periodontal ligament mesenchymal stem cells stimulated with lps-p-gingivalis. Eur. J. Inflamm..

[7] Bakopoulou A., Leyhausen G., Volk J., Koidis P., Geurtsen W. (2012). Effects of resinous monomers on the odontogenic differentiation and mineralization potential of highly proliferative and clonogenic cultured apical papilla stem cells. Dent. Mater..

[8] Di Nisio C., De Colli M., di Giacomo V., Rapino M., Di Valerio V., Marconi G.D., Gallorini M., Di Giulio M., Cataldi A., Zara S. (2015). A dual role for beta1 integrin in an in vitro streptococcus mitis/human gingival fibroblasts co-culture model in response to tegdma. Int. Endod. J..

[9] Pizzicannella J., Gugliandolo A., Orsini T., Fontana A., Ventrella A., Mazzon E., Bramanti P., Diomede F., Trubiani O. (2019). Engineered Extracellular Vesicles From Human Periodontal-Ligament Stem Cells Increase VEGF/VEGFR2 Expression During Bone Regeneration. Front. Physiol..

[10] Maiuolo J., Maretta A., Gliozzi M., Musolino V., Carresi C., Bosco F., Mollace R., Scarano F., Palma E., Scicchitano M. (2018). Ethanol-induced cardiomyocyte toxicity implicit autophagy and nfkb transcription factor. Pharmacol. Res..

[11] Li Y., Wang S., Ni H.M., Huang H., Ding W.X. (2014). Autophagy in alcohol-induced multiorgan injury: Mechanisms and potential therapeutic targets. BioMed Res. Int..

[12] Teti G., Orsini G., Salvatore V., Focaroli S., Mazzotti M.C., Ruggeri A., Mattioli-Belmonte M., Falconi M. (2015). Hema but not tegdma induces autophagy in human gingival fibroblasts. Front. Physiol..

[13] Tanida I., Ueno T., Kominami E. (2008). Lc3 and autophagy. Methods Mol. Biol..

[14] Hosokawa N., Sasaki T., Iemura S., Natsume T., Hara T., Mizushima N. (2009). Atg101, a novel mammalian autophagy protein interacting with atg13. Autophagy.

[15] Kabeya Y., Mizushima N., Uero T., Yamamoto A., Kirisako T., Noda T., Kominami E., Ohsumi Y., Yoshimori T. (2000). Lc3, a mammalian homologue of yeast apg8p, is localized in autophagosome membranes after processing. EMBO J..

[16] Mercer C.A., Kaliappan A., Dennis P.B. (2009). A novel, human atg13 binding protein, atg101, interacts with ulk1 and is essential for macroautophagy. Autophagy.

[17] Jung C.H., Jun C.B., Ro S.H., Kim Y.M., Otto N.M., Cao J., Kundu M., Kim D.H. (2009). Ulk-atg13-fip200 complexes mediate mtor signaling to the autophagy machinery. Mol. Biol. Cell.

[18] Ganley I.G., Lam D.H., Wang J.R., Ding X.J., Chen S., Jiang X.J. (2009). Ulk1 center dot atg13 center dot fip200 complex mediates mtor signaling and is essential for autophagy. J. Biol. Chem..

[19] Kundu M., Lindsten T., Yang C.Y., Wu J., Zhao F., Zhang J., Selak M.A., Ney P.A., Thompson C.B. (2008). Ulk1 plays a critical role in the autophagic clearance of mitochondria and ribosomes during reticulocyte maturation. Blood.

[20] Busca R., Pouyssegur J., Lenormand P. (2016). Erk1 and erk2 map kinases: Specific roles or functional redundancy?. Front. Cell Dev. Biol..

[21] Zara S., De Colli M., Rapino M., Di Valerio V., Marconi G.D., Cataldi A., Macchi V., De Caro R., Porzionato A. (2013). Nf-kappab involvement in hyperoxia-induced myocardial damage in newborn rat hearts. Histochem. Cell Biol..

[22] Ogier-Denis E., Pattingre S., El Benna J., Codogno P. (2000). Erk1/2-dependent phosphorylation of galpha-interacting protein stimulates its gtpase accelerating activity and autophagy in human colon cancer cells. J. Biol. Chem..

[23] Pattingre S., Bauvy C., Codogno P. (2003). Amino acids interfere with the erk1/2-dependent control of macroautophagy by controlling the activation of raf-1 in human colon cancer ht-29 cells. J. Biol. Chem..

[24] Ellington A.A., Berhow M.A., Singletary K.W. (2006). Inhibition of akt signaling and enhanced erk1/2 activity are involved in induction of macroautophagy by triterpenoid b-group soyasaponins in colon cancer cells. Carcinogenesis.

[25] Shinojima N., Yokoyama T., Kondo Y., Kondo S. (2007). Roles of the akt/mtor/p70s6k and erk1/2 signaling pathways in curcumin-induced autophagy. Autophagy.

[26] Anding A.L., Baehrecke E.H. (2017). Cleaning house: Selective autophagy of organelles. Dev. Cell.

[27] Diomede F., Gugliandolo A., Cardelli P., Merciaro I., Ettorre V., Traini T., Bedini R., Scionti D., Bramanti A., Nanci A. (2018). Three-dimensional printed PLA scaffold and human gingival stem cell-derived extracellular vesicles: A new tool for bone defect repair. Stem Cell Res. Ther..

[28] Diomede F., Rajan T.S., Gatta V., D’Aurora M., Merciaro I., Marchisio M., Muttini A., Caputi S., Bramanti P., Mazzon E. (2017). Stemness maintenance properties in human oral stem cells after long-term passage. Stem Cells Int..

[29] Pizzicannella J., Diomede F., Merciaro I., Caputi S., Tartaro A., Guarnieri S., Trubiani O. (2018). Endothelial committed oral stem cells as modelling in the relationship between periodontal and cardiovascular disease. J. Cell. Physiol..

[30] Diomede F., Zini N., Pizzicannella J., Merciaro I., Pizzicannella G., D’Orazio M., Piattelli A., Trubiani O. (2018). 5-aza exposure improves reprogramming process through embryoid body formation in human gingival stem cells. Front. Genet..

[31] Gugliandolo A., Diomede F., Cardelli P., Bramanti A., Scionti D., Bramanti P., Trubiani O., Mazzon E. (2018). Transcriptomic analysis of gingival mesenchymal stem cells cultured on 3d bioprinted scaffold: A promising strategy for neuroregeneration. J. Biomed. Mater. Res. Part A.

[32] Rajan T.S., Giacoppo S., Trubiani O., Diomede F., Piattelli A., Bramanti P., Mazzon E. (2016). Conditioned medium of periodontal ligament mesenchymal stem cells exert anti-inflammatory effects in lipopolysaccharide-activated mouse motoneurons. Exp. Cell Res..

[33] Diomede F., Gugliandolo A., Scionti D., Merciaro I., Cavalcanti M.F., Mazzon E., Trubiani O. (2018). Biotherapeutic effect of gingival stem cells conditioned medium in bone tissue restoration. Int. J. Mol. Sci..

[34] Ballerini P., Diomede F., Petragnani N., Cicchitti S., Merciaro I., Cavalcanti M., Trubiani O. (2017). Conditioned medium from relapsing-remitting multiple sclerosis patients reduces the expression and release of inflammatory cytokines induced by lps-gingivalis in thp-1 and mo3.13 cell lines. Cytokine.

[35] Pizzicannella J., Cavalcanti M., Trubiani O., Diomede F. (2018). Microrna 210 mediates vegf upregulation in human periodontal ligament stem cells cultured on 3dhydroxyapatite ceramic scaffold. Int. J. Mol. Sci..

[36] Diomede F., Zingariello M., Cavalcanti M., Merciaro I., Pizzicannella J., De Isla N., Caputi S., Ballerini P., Trubiani O. (2017). Myd88/erk/nfkb pathways and pro-inflammatory cytokines release in periodontal ligament stem cells stimulated by porphyromonas gingivalis. Eur. J. Histochem. EJH.

[37] Diomede F., Merciaro I., Martinotti S., Cavalcanti M.F.X.B., Caputi S., Mazzon E., Trubiani O. (2016). Mir-2861 is involved in osteogenic commitment of human periodontal ligament stem cells grown onto 3d scaffold. J. Biol. Regul. Homeost. Agents.

[38] Cavalcanti M.F., Maria D.A., de Isla N., Leal-Junior E.C., Joensen J., Bjordal J.M., Lopes-Martins R.A., Diomede F., Trubiani O., Frigo L. (2015). Evaluation of the Proliferative Effects Induced by Low-Level Laser Therapy in Bone Marrow Stem Cell Culture. Photomed. Laser. Surg..

[39] Trubiani O., Guarnieri S., Diomede F., Mariggio M.A., Merciaro I., Morabito C., Cavalcanti M.F., Cocco L., Ramazzotti G. (2016). Nuclear translocation of pkcalpha isoenzyme is involved in neurogenic commitment of human neural crest-derived periodontal ligament stem cells. Cell. Signal..

[40] Pizzicannella J., Rabozzi R., Trubiani O., Di Giammarco G. (2011). Histidine-tryptophan-ketoglutarate solution helps to preserve endothelial integrity of saphenous vein: An immunohistochemical and ultrastructural analysis. J. Biol. Regul. Homeost. Agents.

[41] Rajan T.S., Scionti D., Diomede F., Grassi G., Pollastro F., Piattelli A., Cocco L., Bramanti P., Mazzon E., Trubiani O. (2017). Gingival stromal cells as an in vitro model: Cannabidiol modulates genes linked with amyotrophic lateral sclerosis. J. Cell. Biochem..

[42] Giacoppo S., Thangavelu S.R., Diomede F., Bramanti P., Conti P., Trubiani O., Mazzon E. (2017). Anti-inflammatory effects of hypoxia-preconditioned human periodontal ligament cell secretome in an experimental model of multiple sclerosis: A key role of il-37. FASEB J..

[43] Mammana S., Gugliandolo A., Cavalli E., Diomede F., Iori R., Zappacosta R., Bramanti P., Conti P., Fontana A., Pizzicannella J. (2019). Human gingival mesenchymal stem cells (gmscs) pre-treated with vesicular moringin nanostructures as a new therapeutic approach in a mouse model of spinal cord injury. J. Tissue Eng. Regen. Med..

[44] Trubiani O., Toniato E., Di Iorio D., Diomede F., Merciaro I., D’Arcangelo C., Caputi S. (2012). Morphological analysis and interleukin release in human gingival fibroblasts seeded on different denture base acrylic resins. Int. J. Immunopathol. Pharmacol..

[45] Wang N., Zhang Q., Luo L., Ning B., Fang Y. (2018). Beta-asarone inhibited cell growth and promoted autophagy via p53/bcl-2/bclin-1 and p53/ampk/mtor pathways in human glioma u251 cells. J. Cell. Physiol..

[46] Waldner M., Fantus D., Solari M., Thomson A.W. (2016). New perspectives on mtor inhibitors (rapamycin, rapalogs and torkinibs) in transplantation. Br. J. Clin. Pharmacol..

